# Supplementary material for: Are carbon emissions trading and green financial instruments synergistic? -Comprehensive quantitative research based on content analysis
Source: PLoS One. 2024 Mar 7;19(3):e0298601. doi: 10.1371/journal.pone.0298601 (PMC10919601; doi:10.1371/journal.pone.0298601)
Supplement: S1 Data — (ZIP) [file pone.0298601.s001.zip › Data/地级市Z_F工作报告.zip!/╔┬╬≈/▒a╝a╩╨/└┤╫╘╣1⁄2╓┌║┼í╛┬φ┐╦╩2╛▌═°í┐_2011.docx]

各位代表：

现在，我代表市人民政府向大会作工作报告，请连同《宝鸡市国民经济和社会发展第十二个五年规划纲要(草案)》一并审议，并请市政协委员和其他列席人员提出意见。

一、去年和“十一五”工作回顾

2010年，是“十一五”圆满收官之年，也是我市经济社会发展取得重大成就的一年。一年来，市政府认真贯彻中央和省、市委一系列决策部署，以科学发展观为指导，抢抓关天规划和新一轮西部大开发实施机遇，坚持“四个第一”，推进“五大突破”，强力实施“率先发展八大工程”，全面完成了市十三届人大四次会议确定的各项目标任务。全年实现地方生产总值976.1亿元，增长14.4%；财政总收入达到106.3亿元，增长22.3%，其中地方财政收入38.8亿元，增长28.7%；城镇居民人均可支配收入和农民人均纯收入分别达到18978元、5040元，增长16.1%和20.4%。

重大项目建设进展顺利，固定资产投资快速增长。“率先发展八大工程”确定的197个重点项目，完成投资410亿元；全年完成全社会固定资产投资835.2亿元，增长30.7%；实现合同引资645.7亿元，到位资金266.6亿元；争取中省预算内资金13.8亿元，居全省前列。产业项目建设取得重大突破，陕汽30万辆微型车、法士特二期铝压制品、中铁宝桥道岔扩能、千阳海螺水泥、冀东水泥凤翔生产线等项目建成投产，宝石机械异地扩建、国核锆材、150万吨甲醇、汉德车桥扩建技改、通力专用汽车等项目进展顺利。重大基础设施项目强力推进，宝平高速千阳至陇县段建成通车，西宝高速改扩建和西宝客运专线建设进展顺利，麟北运煤铁路专线开工建设，渭河南岸铁路枢纽专线等项目前期工作进展良好，大城市综合交通网络正在形成。

大企业大集团快速成长，工业经济实力显著提升。加速培育大产业集群，全市规模以上工业实现增加值441.5亿元，增长19.9%。12户大企业大集团完成工业总产值662.3亿元，宝钛集团成为第三个百亿企业，东岭集团在中国企业500强中前移28位，秦川、宝桥、西凤酒等企业保持了40%以上的增速。大力推进产学研合作，宝石钢管石油天然气管材工程技术研究中心成功进入国家级行列，省级以上企业（工程）技术中心达到36个，被命名为国家创新型试点城市、国家知识产权示范城市。高新区实现地方生产总值300亿元，增长25%，跻身国家创新型科技园区；蔡家坡开发区汽车及零部件制造基地建设迈出新步伐。县区工业园承载能力不断增强，34户企业成功入园，园区已成为引领县域工业发展的引擎。市属国有企业改革实现突破，宝鸡机床集团、西凤酒股份公司增资扩股、宝鸡制药机械厂整体改制顺利完成，红旗民爆上市进入辅导期，陕西九棉公司政策性破产进入法律程序。

大城市建设加力提速，人居环境更加优越。新的城市总体规划得到省政府批准，南客站片区和秦岭北麓生态新城区开发加快推进。冯家山引水复线、5万吨中水回用、老城区集中供热改造、高速客运站等工程顺利完工。东仁堡旧城改造、代家湾城中村改造进展顺利。石头河引水、蟠龙塬供水、引红济石等水利工程启动实施。持续推进“绿色宝鸡”建设，全面启动陕西西大门绿化。深入开展创建国家生态县区、生态乡镇和生态村活动，国家生态示范市通过考核验收，在西北地区率先形成生态示范城镇群。凤翔、麟游、千阳、凤县跻身国家园林县城，岐山、眉县荣获省级园林县城，率先实现了省级园林县城全覆盖。节水型社会建设试点工作扎实推进，各县污水处理厂、垃圾处理场全部建成投用，节能减排“两项任务”圆满完成。

“三农”工作扎实推进，县域经济活力显现。畜、粮、果、菜等特色优势产业加快发展，现代牧业两万头奶牛场和10个千头奶牛场顺利建成，奶牛标准化养殖“千阳模式”在全省推广；全年粮食总产171.5万吨，连续七年获得丰收，扶风县荣获全国粮食生产先进县；新建果园21万亩、核桃标准园16.9万亩，眉县被评为全国果菜标准化建设十强县；发展设施蔬菜2万亩，太白—凤县秦岭蔬菜带成为全省最大的高山绿色有机蔬菜生产基地。全年新增市级以上农业产业化龙头企业50户。投资25亿元，实施了农村安全饮水、通村公路、信息入户、环境综合整治等工程，农村生产生活条件进一步改善。突破西山战略取得阶段性成效，集中连片特殊区域扶贫开发持续推进，全市8.6万名农村贫困人口实现脱贫。积极开展“晋星创建”活动，全市四星村、五星村分别达到376个和131个。凤县跨入“陕西十强县”，凤翔、岐山入围“中国最具投资潜力中小城市百强县”，太白、麟游、千阳荣获全省县域经济社会发展争先进位奖。全市集体林权制度主体改革提前一年完成，太白县跻身全国林改百县典型。制定出台了农村宅基地和承包地退出补偿等政策，5万农村居民进城落户，试点工作初见成效。

第三产业快速发展，消费市场日趋活跃。成功举办了第十届“两节一会”和“翰墨石鼓”书法名家邀请展、“九成宫杯”全国书法大赛等重大活动，代表陕西参加了第六届深圳文博会，取得良好成效。宝鸡青铜器博物院建成开放，金台观文化景区、西府古镇、石鼓山区域开发等项目进展顺利，周秦文化产业示范区成为国家级示范基地，中华礼乐城、岐山周公庙、凤县凤凰湖等景区通过国家4A级景区初验。全市旅游综合收入突破百亿元大关，增长28.6%。编制了全市物流业发展规划，陈仓物流园区建设进展顺利，嘉隆国际商城、新冠森家具建材城等项目建成投用，成功引进了红星美凯龙、大润发等一批知名商贸企业。全年实现社会消费品零售总额304.6亿元，增长18.4%。全市实现新增贷款104亿元，有力支持了经济发展。

民生得到持续改善，社会事业全面发展。实施民生工程项目47类，完成投资36.6亿元。全面推行了城镇居民养老保险、被征地农民就业和社会保障制度、城乡居民临时救助制度，提高了城乡低保、廉租房租金补贴等8项标准，新型农村养老保险45岁以上人员参保率达到91.7%，年内新建廉租住房5737套28.7万平方米。凤县实现城乡统一的居民养老、医疗保障制度。全面建成了“三级管理、四级服务”的就业保障服务网络，全年新增城镇就业5万人，凤翔县获全国劳动力转移就业工作示范县。成功应对“7.18”、“7.23”洪涝灾害，紧急转移安置受灾群众3.1万人，重建倒塌房屋2522户，维修房屋11826户。坚持优先发展教育，教育强县占到全省总数1/4，高考二本上线万人比为全省第一，教育资助受惠学生51.2万人次，创历史之最。金台区建成省级教育强区，太白县建成省级“双高普九”县，凤县、太白实现高中阶段免费教育，宝鸡职业技术学院入围省级示范性高职院校行列，在全省率先通过国家二类城市语言文字工作评估验收。强化食品药品监管，推进“健康宝鸡”行动，公立医院改革试点工作进展良好，国家基本药物“零差价”制度和药品“三统一”顺利实施。岐山、扶风、陇县、太白建成国家卫生县城，实现国家卫生县城全覆盖。市文化广电中心开工建设，市直文化单位体制改革基本完成。实现省级计生优质服务先进县全覆盖，被授予全国人口计生综合改革和婚育新风进万家活动示范市。体育事业蓬勃发展，承办了全国散打冠军赛等六项大赛，参加省十四届运动会取得赛会第一名好成绩，女子游泳、链球选手分别获得广州亚残会金牌和亚运会银牌。荣获全国人民防空先进城市，通过全国双拥模范城“七连冠”省级验收。关心下一代工作受到全国表彰。

社会管理全面加强，民主法治建设不断深化。广泛开展“五五”普法，积极排查调处矛盾纠纷，一批信访突出问题得到解决。扎实推进最安全城市创建活动，深入开展严打专项斗争，加强学校、幼儿园及周边环境整治，平安宝鸡建设取得新进展，群众对社会治安的满意率连续七年保持在90%以上。严格落实安全生产责任制，安全生产形势总体平稳，陇县创建为国家平安畅通县。加强民主法制建设和政府自身建设，自觉接受市人大、市政协监督，广泛听取人大代表、政协委员、民主党派以及专家学者的意见建议，认真办理人大代表和政协委员议案、提案。法制政府建设扎实开展，行政行为不断规范。市县机构改革和撤乡并镇任务基本完成，机关办事效率和为民服务水平进一步提高。与此同时，监察、审计、统计、物价、气象、档案、地震、地志、民族宗教、国家安全、妇女儿童、侨务外事、扶残助残、慈善老龄等各项工作也取得了新的成绩。

2010年，我市经济社会发展取得的优异成绩，为“十一五”画上了圆满句号。“十一五”时期，面对国际金融危机的严重冲击，面对冰雪、地震、洪涝等自然灾害，面对十分繁重的经济社会发展任务，面对改革发展稳定中存在的困难和问题，市政府坚持科学决策，紧盯大事不放，着力破解发展难题，带领全市人民团结奋进、苦干实干，共同谱写了宝鸡历史上的光辉篇章。五年来,我们在危机中抢抓机遇，大上项目，上大项目，发展后劲显著增强，综合实力跃上新的台阶。生产总值年均增长14.6%，人均生产总值接近4000美元；全社会固定资产投资累计完成2470.7亿元，年均增长37%，实施10亿元以上大项目43个，总投资1480亿元。财政总收入、地方财政收入分别是2005年的2.6倍和3.2倍；城镇居民人均可支配收入、农民人均纯收入均翻了一番多；跻身全国综合实力百强城市、“新中国60年60个城市发展代表”行列。五年来，我们围绕特色优势产业，狠抓百亿元大企业大集团建设，持续推动产业集群和工业园区发展，工业经济取得新的突破。大企业大集团成为引领工业经济的龙头，22户企业销售收入超过10亿元，3户企业过百亿；全市规模以上工业总产值突破千亿元大关，是2005年的3.2倍。五年来，我们坚持规模扩张和功能提升并举，突出重大基础设施和生态环境建设，狠抓城市管理，大城市建设实现新的跨越。“四横十纵一环一道”的大城市交通骨架初步形成，市区建成区面积达到93平方公里，是2005年的1.6倍；建成区人口85万，城镇化水平提高5个百分点。城市道路通讯、绿化环卫、水电气暖、商业网点、教育卫生、文化体育、社区服务等公共服务设施逐步完善，荣获中国人居环境奖、国家节水型城市、国家森林城市、全国绿化模范城市、全国环境优美城市、中国十大生态宜居城市、中国金融生态城市、中国最具国际影响力的旅游城市等多项荣誉。五年来，我们加大财政投入，强化支农惠农政策，不断深化农村改革，创新体制机制，县域经济发展呈现新的活力。农业特色产业基地规模不断扩大，形成了“畜牧为主导、果菜为特色、粮油为基础”的现代农业发展新格局；县域经济整体实力明显增强，占全市经济的比重达到42.5%，陈仓区、凤翔县、凤县、渭滨区分别进入全省十强县、五强区行列；非公有制经济快速发展，比重由43.3%提升到48.3%。五年来，我们坚持以人为本，大力推进民生工程建设，创新社会保障制度，使老百姓得到更多实惠，人民生活得到新的改善。新型农村养老保险在全国创出了宝鸡模式，社会保障体系实现制度上的无缝隙全覆盖；“民生八大工程”累计完成投资108.9亿元；实施了645个社会福利、社会救助、城乡社区等项目，有力保障了30多万特殊困难群众基本生活；在全省首家实施了中小学生“蛋奶工程”，教育资助体系实现全覆盖；全面完成乡镇卫生院建设和村卫生室标准化改造，母亲健康工程使57万农村育龄妇女得到免费体检和医治；完成移民扶贫搬迁7.8万人，35万人摆脱贫困，解决了95万人的安全饮水问题。

各位代表，“十一五”的五年，是我们抢抓机遇、攻坚克难、创新实干的五年，是基础设施显著改善、社会事业蓬勃发展、群众生活质量明显提高的五年，是宝鸡历史上发展速度最快、经济质量最好、综合实力提升幅度最大的五年，为“十二五”更好更快发展创造了良好条件。这是市委科学决策、正确领导的结果，是市人大、市政协及社会各界大力支持、共谋发展的结果，是全市广大干部群众同心同德、顽强拼搏的结果。在此，我代表市人民政府，向给予我们大力支持和监督的人大代表、政协委员、各民主党派、工商联、各人民团体、社会各界人士，向驻宝单位、解放军和武警部队，表示崇高的敬意和衷心的感谢！

在充分肯定成绩的同时，我们也要正视发展中面临的困难和问题。主要是：大企业大集团数量不多，特色优势产业集群尚未形成，非公经济规模不大，经济结构不尽合理，整体经济实力依然不强；重大基础设施建设相对滞后，大城市建设和城镇化进程缓慢，县域经济总量偏小，统筹城乡发展的难度仍然很大；适应经济、社会转型的体制机制没有完全建立，在城乡就业、居民增收、社会管理等方面还需要进一步加强；公务员队伍的整体素质和工作作风，与加快宝鸡对外开放、满足群众新期望还有一定差距。对于这些问题，我们必须高度重视，努力加以解决。

二、“十二五”的总体思路和战略目标

“十二五”时期，是宝鸡发展极其重要的五年，宝鸡将进入以经济转型为前提的科学发展提速期、以社会转型为基础的全面小康攻坚期、以体制转型为核心的统筹发展关键期。统筹考虑国内外发展环境的新变化、新特点，深刻分析“十二五”时期全市面临的机遇和挑战，发展机遇与挑战并存，但机遇大于挑战，仍处在加快发展的黄金期，我们必须坚定信心、抢抓机遇，比拼争先、积极作为，为实现全面小康目标奠定坚实基础。

“十二五”期间，宝鸡经济社会发展的基本思路是：以邓小平理论和“三个代表”重要思想为指导，全面贯彻落实科学发展观，坚持“四个第一”、推进“五大突破”，抢抓关天规划实施和新一轮西部大开发的重大机遇，以转变发展方式为主线，加快经济结构战略性调整，深入实施科技创新和人才战略，不断深化体制机制改革，扎实推进城乡统筹发展，着力加快基础设施建设、提高发展保障能力；着力培育产业集群、增强经济综合实力；着力加快大城市建设、构建关中西部城镇群；着力改善生态环境、创建最佳宜居城市；着力深化农村改革、推进城乡一体化发展；着力保障和改善民生、推动社会和谐稳定，努力打造关天副中心、建设和谐新宝鸡。

打造关天副中心、建设和谐新宝鸡，是“十二五”期间全市工作的主题。要按照“实力宝鸡、创新宝鸡、开放宝鸡、人文宝鸡、生态宝鸡、幸福宝鸡”的目标要求，在经济发展上，使宝鸡在关天经济区中除西安之外，经济总量最大、工业实力最强、城乡居民收入最多，成为国家先进装备制造业基地、新材料研发生产基地、全省统筹科技资源改革示范区，综合实力进入西部中等城市前10位；在大城市建设上，城市规模快速扩张，综合交通网络基本形成，生态环境更加优美，成为西部重要的交通枢纽、现代化生态园林大城市、陕甘川宁毗邻地区的区域性中心城市；在社会建设上，社会事业发展、民生保障水平、平安社会建设走在西部前列，成为统筹城乡发展的示范区。

主要预期目标是：到2015年，实现地区生产总值、人均生产总值、财政总收入、地方财政收入、城镇居民人均可支配收入、农民人均纯收入“六个翻番”。地区生产总值达到2000亿元，年均增长13%以上；经济总量进入西部中等以上城市前列，人均生产总值超过全省平均水平；固定资产投资年均增长25%，累计投资超过8500亿元；全社会研发投入占GDP的比重达到3%；城市建成区人口达到115万人，面积达到110平方公里，城镇化率达到53%；绿化覆盖率、森林覆盖率分别达到45.5%和55%以上，单位GDP能耗比2010年下降16%；社会事业主要指标位居全省前列，社会繁荣稳定、公平正义、文明和谐。

实现上述目标，必须高度重视经济结构战略性调整，积极实施科技创新和人才战略，不断深化体制机制改革，扎实推进城乡统筹发展。经济结构战略性调整，就是要提高县域经济、非公经济在全市经济中的比重，大力发展外向型经济、第三产业和现代农业，使经济结构不断适应发展方式的转变；实施科技创新和人才战略，就是要加快企业研发中心建设，积极引进高端人才，加强产学研结合，使科技创新成为转变发展方式的重要支撑；深化体制机制改革，就是要建立健全投融资体制，创新园区管理和经营机制，深化公共服务等重点领域改革，使体制机制创新成为转变发展方式的强大动力；推进城乡统筹发展，就是要加快城乡基础设施建设，推进制度创新，缩小收入差距，让城乡居民共享发展成果，大力支持创业就业，增加城乡居民收入，提高社会保障水平，使保障和改善民生成为转变发展方式的出发点和落脚点。“十二五”期间，要着力完成六个方面的战略任务：

基本完善重大基础设施。按照科学的规划理念，前瞻性做好交通、水利等重大基础设施规划和建设，构建功能配套、安全高效的现代化基础设施体系。构筑城乡综合交通网络，进一步巩固提升宝鸡的交通枢纽地位。完成渭河综合整治工程，建设一批骨干水利工程、大中型水库及城市水源工程，进一步完善水利基础设施。实施物联网工程，推动基础设施的信息化和智能化，争取把宝鸡建成全国信息网络的重要节点。加强市区、县城、重点镇、重点园区道路、供排水、供暖供气、污水垃圾处理等公用设施建设，实现市政公共设施基本配套到位。

初步形成优势产业集群。围绕特色优势产业，倾力打造10户以上百亿企业集团。培育壮大汽车及零部件、钛及钛合金、机床工具、石油装备、煤化工、酒类、轨道交通、航空安全装备等优势产业集群，建成全国先进装备制造业基地和以“宝鸡?中国钛谷”为主的新材料研发生产基地，形成特色鲜明、优势突出的现代工业格局。到“十二五”末，形成千亿元产业集群2个、五百亿元产业集群2个，全部工业增加值超过1000亿元。科技创新水平居西部前列，在全省率先建成国家创新型城市。

建成区域性中心大城市。科学拓展城市发展空间，向东重点建设高新三期、科技新城、陈仓物流园区，向南重点发展南客站片区和秦岭北麓现代化生态功能区，向北重点开发蟠龙塬新区。加快蔡家坡副中心建设，积极推进凤翔撤县设区，加大县城和重点镇建设，着力构建关中西部城镇群。合理布局学校、医院、文化、商业、餐饮等公共服务设施，实施“数字化、精细化、人性化”城市管理，提升公共服务功能和管理水平。打响炎帝故里、佛骨圣地、秦岭中央公园、周秦文化产业示范区等文化旅游品牌，建成区域性商贸物流中心和文化旅游中心。

创建西部一流生态环境。继续推进节能减排，加快绿色宝鸡建设，倡导低碳经济和循环经济，在西部率先建成国家生态市，使宝鸡既有园林城市，又有秀美乡村，四季常青，三季有花，投资环境明显改善，政府管理和公共服务水平持续提升，成为投资、居住两相宜的活力宝鸡、魅力城市。

实现统筹城乡协调发展。从城乡规划、产业发展、基础设施、公共服务、劳动就业、社会管理六个方面加大统筹力度。改革城乡土地管理、社会保障、户籍管理，加快推进公共服务均等化，引导有条件农民向市区、县城和重点镇集中，力争每年向城镇转移农民10万人，形成城乡互融、体制协调、共同繁荣的良好局面。到2015年，把渭河川道区域建成统筹城乡发展的重点示范区。

持续提高人民生活水平。健全就业创业服务体系，千方百计增加城乡居民收入；加大保障性住房建设力度，实现居者有其屋；不断增加财政对教育的投入，进一步促进教育公平；继续加强医疗卫生服务体系建设，实现公共卫生服务城乡一体化；加快文化体育事业发展，满足人民群众不断增长的精神需求；完善社会保障和社会救助体系，建立大社保“宝鸡模式”，实现由“解困民生”到“普惠民生”的历史跨越。

三、2011年的政府工作

今年，是实施“十二五”规划的第一年，也是市委确定的“转型突破年”。政府工作要围绕“十件大事”，推动“五大突破”向更高层次迈进，确保“十二五”发展稳健起步、良好开局。全市经济社会发展的主要预期目标是：生产总值增长14%，财政总收入、地方财政收入均增长17%，城镇登记失业率控制在4%以内，居民消费价格总水平涨幅控制在4%左右，城镇居民人均可支配收入和农民人均纯收入均增长15%，全社会固定资产投资增长25%以上，万元GDP能耗下降3.5%，主要污染物排放指标控制在规定范围以内。具体做好八个方面的工作：

（一）壮大特色优势产业集群，推动工业转型升级

支持大企业大集团快速扩张。支持龙头企业就地技改扩能，推动土地、资金等生产要素向大企业聚集。积极实施陕汽30万辆微型车二期工程及汽车零部件配套项目，推动陕汽扩张规模；实施核级锆材生产线、钛材加工等项目，把宝钛集团打造成世界一流钛加工企业；加快宝石机械技改搬迁项目建设，巩固行业领先地位；支持秦川集团开展区域产业整合、并购重组，建设国内精密数控机床研发制造一流企业；抓好西凤酒集团扩建技改、宝二电二期扩建、徐矿集团150万吨甲醇、西电宝光铁道电器生产线、宝成航空精密制造工业园等项目建设。开工法士特锻造二期、陕西金鼎15万吨铸造等项目。进一步完善大企业培育体系，扶持高成长性重点企业提速发展，形成梯次跃进的大企业团队。

做大做强九大产业集群。市财政每年安排5000万元专项资金，扶持龙头企业、产业链配套企业发展。支持龙头企业就近就地开展配套协作，建立龙头企业与配套企业的战略联盟，延长产业链，推动产业转型升级，确保全市工业增速达到17%，实现工业增加值605亿元。突出抓好市属困难企业脱困工作，加快推进商贸流通企业和外贸企业改制重组。组建国有资产经营公司，推进企业重组和国有资本运营。支持红旗民爆实现上市，西凤酒股份上市取得实质性进展。

加快工业园区发展步伐。支持高新区建设国家创新型科技园区，蔡家坡开发区晋级为国家级开发区，凤州、长青、绛帐等基础较好的园区进入省级开发区行列。积极探索县区工业园由国家级、省级园区代管等开发管理模式，赋予县区工业园更大的自主权。做大做强凤县黄金、铅锌产业，着力打造关天经济区生态型铅锌产业基地。加快眉县砖机制造产业发展，打造中国砖机城。

大力推进科技创新。全力推进国家级创新型试点城市建设，支持大中型企业建立国家级或省级工程技术中心。实施一批重大科技专项，攻克关键技术，开发一批新产品。加大统筹科技资源力度，落实省市科技会商制度，开展科技进园区活动。以企业为主体，深化产学研合作，建立企业、高校和科研院所有机结合的产业技术创新机制，促进科技成果转化。建立更加灵活的人才培养、引进、使用机制，大胆引进一批创新型管理人才、高技术领军人才，满足经济社会发展需要。把高新区作为宝鸡创新型城市建设的核心示范区，汇聚创新资源，推动产业聚集，打造现代科技生态新城。办好国家级高新区主任联席会议。

推动非公经济快速发展。出台我市非公经济发展的配套政策，营造非公经济快速发展的宽松环境。大力发展加工贸易、社区服务、农产品加工等劳动密集型产业，引导和支持非公经济、中小企业围绕产业集群开展协作配套。发挥产业政策和行业规划的导向作用，引导非公经济投资高新技术产业、现代服务业和现代农业，扩大非公经济总量，年内非公经济占到经济总量的49%。

大力发展新兴产业。以宝成仪表、凌云电器等骨干企业为龙头，建设高水平的中国西部航空安全装备产业园。以宝桥、南车集团等企业为龙头，大力实施高速铁路道岔、接触网器材等项目，发展轨道交通装备产业。以徐矿集团、烽火通信、长岭科技、秦川集团、宝石机械等企业为龙头，加快太阳能电池及组件、精密数控风电专用机床、大功率风电机组生产线等项目的实施，促进新兴产业加快发展。

（二）实施谋划一批重大项目，保持固定资产投资强劲增长

全力抓好重大项目建设。今年，安排市级重点项目100个，总投资1500亿元，年度投资400亿元。积极破解影响项目建设的突出问题，增加土地储备，搞好占补平衡，确保项目建设用地。完善投融资体制，创新金融品种，发挥上市公司融资作用，鼓励民间资本投资，采取多种方式，解决资金不足问题。积极做好项目前期工作，强化项目建设服务保障，实行重大项目领导包抓制度，为项目建设创造良好环境。

积极谋划争取项目。紧跟国家投资政策，围绕关天规划、“十二五”发展规划和全面建设小康社会目标，量化分解指标任务。重点在保障性住房、教育、卫生、养老、生态等方面，谋划包装项目，积极争取国家投入。做好重大项目的策划包装，加大争取力度，力争宝成铁路复线、关中城际铁路、城市轻轨、岐蔡复线、银洞峡水库等一大批重大基础设施项目，挤进国家和省上盘子。

加大招商引资力度。围绕九大产业集群，着力引进一批配套企业和项目。发挥园区招商的主阵地作用，按照托管共建、利益共享的原则，主动加强与江苏等东部地区工业园区的合作与联系。坚持把招商引资作为“一把手”工程，把招商绩效与目标考核、干部使用挂钩。继续优化投资环境，搞好全方位服务，提高办事效率，吸引一大批外地企业落户，年内实现合同引资550亿元，到位资金230亿元。

（三）构建现代农业产业体系，促进农民持续增收

夯实农业农村发展基础。启动新一轮农村电网改造升级工程，强化电力保障。抓好县乡公路改造，完善通村公路安保设施，确保农村公路安全畅通。深入持续突破西山，大力实施整村推进、贫困户安居等“六大扶贫工程”，建立农村最低生活保障和扶贫开发政策的有效衔接制度，对农村低收入人口全面实施扶贫，带动三大区域13个重点板块加快扶贫开发，解决6万农村贫困人口的脱贫。继续推进晋星村创建，年内五星村、四星村分别达到160个和446个。

加快水利改革发展。认真贯彻中央1号文件，加强农村水利基础设施建设。加大水土保持和水生态保护力度，合理开发水能资源。深化水权制度改革，严格水资源管理，建设节水型社会。年内建设各类农村饮水工程200处，解决20万农村人口的饮水安全问题，完成7座病险水库的除险加固，发展节水灌溉面积15万亩，治理水土流失面积300平方公里。

积极发展现代农业。大力实施国家千亿斤粮食生产能力工程，加快建设渭北塬区百万亩优质苹果、百万亩核桃、百万亩蔬菜、秦岭西山百万亩花椒和60万亩猕猴桃基地，年内新增苹果13万亩、猕猴桃7万亩、设施蔬菜2万亩。加快千阳和陇县奶牛标准化养殖示范县、凤翔设施蔬菜示范县建设，推进扶风等县区百万头生猪大县建设，提升现代农业发展水平。大力发展农业产业化龙头企业，使亿元以上龙头企业达到20户。加强农业标准化和农产品质量安全工作，扩大无公害农产品、绿色食品和有机食品的生产规模，创建畜禽标准化示范场20个。抓好宝鸡农产品物流中心、眉县猕猴桃物流园区建设，完善农产品销售市场体系。支持高校、科研院所同农民专业合作社、龙头企业、农户开展多种形式的技术合作，促进农业科技成果转化。

拓宽农民增收渠道。千方百计抓好农民增收，实现经济发展与农民收入同步增长。启动实施“专业农户成长计划”，提高农民组织化程度和农产品市场竞争力，挖掘农业内部增收潜力，增加农民经营性收入。加快发展劳务经济，强化农民教育培训，增加农民工资性收入。整合财政支农资金，加大“三农”投入，落实各项强农惠农政策，增加农民转移性收入。年内力争农民人均纯收入达到5800元。

完善农村发展体制机制。积极推进农村产权制度改革，完善各种生产要素流转市场。在依法、自愿、有偿和加强服务的基础上，采取土地银行、承包租赁等多种形式，发展适度规模经营。深化农村金融改革，加快农村金融网络建设，开展土地承包经营权、林权、房屋产权抵押等金融服务，进一步拓宽农业农村发展的融资渠道。搞好第九次村民委员会换届选举。

（四）构筑大旅游发展格局，带动第三产业加速发展

提升文化旅游产业发展水平。加快法门寺文化景区二期、炎帝陵景区、金台观道教文化景区等项目建设进度，开工建设通天河景区专线公路、关山草原景区提升等项目，做大做精周公庙景区，力争法门寺成功创建国家5A级旅游景区。全面推进周秦文化产业示范区建设，抓好石鼓山—茵香河园区开发。加大北首岭、雍城、周原、周公庙、九成宫等大遗址和千湖湿地公园的保护开发，争取保护规划早日得到国家批准。抓好凤县古羌文化旅游产业示范区、宝鸡民俗博物馆、西岐民俗园、关中风情园、六营民艺村等项目建设，加快旅游纪念品商场、中华石鼓园、太白山景区游客服务中心等服务体系建设。办好法门寺国际文化旅游节、太白山旅游登山节和全球华人省亲祭祖大会。年内接待游客突破2000万人次，旅游综合收入增长28%。

扩大商贸流通业规模。不断提升东高新区、代马新区等区域商贸服务功能，完善中心城区市场网络建设。依托“万村千乡市场工程”、“双百市场工程”、“新网工程”、“农超对接”等项目，实施“乡镇超市工程”，培育一批配送能力强、辐射面广的商贸流通企业，完善农村流通市场体系。扎实推动“家电下乡、以旧换新”工作，扩大城乡消费。建立价格调节基金制度，健全完善市场运行监测系统，加大市级储备，全力保障市场供应和物价平稳。

强力推进物流产业发展。运用现代物流管理方式和信息技术改造提升物流业，建设全市统一的物流公共信息平台。完成陈仓物流园区一期建设，启动二期工程，打造综合型物流园区。扶持西部物流、华誉物流等企业发展，培育一批现代物流龙头企业，促进第三方物流发展。推进铁路、公路及航空多式联运的有效链接，构建经济合理的物流交通网络。

大力发展现代服务业。引进各类金融机构，支持各县区组建小额贷款公司、农村资金互助社、融资性担保机构，积极发展村镇银行、农村商业银行，多渠道解决中小企业和“三农”贷款难问题。选择优势企业与深圳、上海创投公司和基金公司开展合作，加快城市融资平台建设。扶持工业设计、技术研发、信息咨询、商务会展等生产性服务业发展，引导装备制造业向服务环节延伸。大力发展中介、创意、服务外包等新兴服务业，积极发展家政、养老、文化、体育、休闲等生活性服务业，提高服务业的附加值和发展水平。

（五）加快推进大城市建设，提升辐射带动能力

抓好重大基础设施项目。加快西宝快速铁路客运专线、宝汉高速、西宝南线改造等项目建设进度，确保宝平高速、西宝高速改扩建等工程建成通车。开工建设宝兰铁路客运专线、降帐至法门寺连接线、西宝北线改造、合凤高速等项目。做好支线机场、环城高速、西宝中线升级改造、渭河南岸铁路枢纽专线、宝中铁路复线等项目前期工作，争取早日开工。加快石头河引水、引红济石调水、宝天750千伏输变电、宝鸡热电联产等项目建设，完善重大基础设施体系。

提高新城区规划建设标准。适应城市化快速发展的新形势，全面提高城市“红线、绿线、蓝线”和地下停车场、小区服务设施等规划标准，严格城市规划管理，认真执行社区服务中心、学校、幼儿园、医院、商业网点等布局规划。加快南客站、代家湾、陈仓物流园、科技新城、虢镇城区等片区开发建设。南客站片区主要建设站前大道、车站广场、高新二小、安置小区等项目；代家湾片区重点实施光明村、代家湾村城中村改造，建设蟠龙塬供水、上塬路、商业文化中心、橡胶坝、配水管网改扩建等工程；陈仓物流园片区重点加快道路、供水、排污等基础设施建设；虢镇城区完善载体服务功能，开工建设北新路等基础设施工程。加强人民防空建设，开发地下空间，完善城市功能。重大建设工程、高层建筑，必须按照地震安全性评价结果进行抗震设防。

完善老城区载体服务功能。投资2亿元，抓紧实施旧城区基础设施改造提升工程，重点抓好公园路立交、宝十路改造、经二路片区市场等49个项目，有效缓解市场、停车场、绿地广场、环卫设施不足和道路不畅等问题。完成代家湾地区和新福路排水管网、高新区二期供水等工程，实施天然气三期利用工程，群众路和姜城、神农、福临堡地区供热工程，提高老城区供气供热保障能力。坚持公交优先发展战略，加快公交场站建设，调整和延伸公交线路，方便群众出行。

提升城市管理水平。建设城市地理信息系统，完善数字化城市管理系统功能，推进城市规划管理信息网和市政公用事业服务信息网建设，实现城市管理全天候、全覆盖、高效化。加强城市规划监督管理，严格规划执法，切实维护规划的严肃性。持续加强城市绿化工作，巩固园林环卫体制改革成果，延伸管理区域，提高管护水平，着力建设干净整洁、文明有序的城市环境。

（六）着眼城乡一体化发展，在统筹城乡上实现突破

高标准建设县城和重点镇。支持县城开展各项创建活动，加快县城和重点镇道路、排水、供气、供暖、绿化、亮化等基础设施建设，聚集发展要素，提升发展水平。按照现代化新城区的标准加快县城建设，使其成为以特色产业、商贸服务和文化旅游等为主的地域性经济、文化和政治中心，辐射带动周边城镇发展。强力推进12个省级重点镇建设，加快县域城镇化步伐。

积极推进有条件农民进城落户。制订农民进城规划，完善相关配套办法，对进城落户的农民，按照“落户条件从宽、办理程序从简、惠及转户居民”的要求，健全户籍、住房、就业、社保、教育、医疗、计生等保障机制。对选择城市居住的农民，继续落实好原有的农村各项社会保障政策，进一步落实责任，完善机构，切实为农民进城落户搞好服务，力争全年完成农村居民进城落户10万人。

扎实开展统筹城乡试点工作。以高新区和蔡家坡开发区为重点，积极探索在人口密集、工业基础好、有一定条件的渭河川道统筹城乡发展的新路径；在凤翔县探索农业比重大、农村人口多、城镇化进程慢的渭北塬区统筹城乡发展的新路径，支持紫荆山新城、长青苑小区加快建设统筹城乡示范点；在凤县、西部山区探索地域广、人口少、生态保护任务重的山区统筹城乡发展的新路径。各县区确定1—2个基础较好、有代表性的试点区域，探索符合实际的统筹城乡发展路子。

（七）更加注重保障和改善民生，全面提高公共服务水平

积极推进创业就业。认真落实支持创业、扩大就业的一系列政策措施，完善政策扶持、创业培训、指导服务三大平台，加快建设创业孵化基地，搞好创业培训和跟踪指导服务，创建全国创业型城市。深入推进人人技能工程，开展创业就业示范创建活动，加大公益性岗位开发力度，对就业困难人员实施就业援助。强化基层就业服务体系建设，普遍推行工资集体协商制度，构建和谐劳动关系，促进充分就业和社会稳定。

继续完善“大社保”体系。将新增财力优先用于民生支出，创造条件建立城乡统一的社会保障制度。深化医疗保险制度改革，建立城乡居民医疗保险市级统筹机制；整合人社、卫生、民政部门经办机构，建立城乡统一的医疗保险新体制；逐步推进新型农村养老保险和城镇居民养老保险并轨，形成统一的城乡居民养老保险制度；试行建立城乡居民大病统筹制度，使城乡居民和干部职工享受同等的大病救助待遇。将劳动就业、社会保险、社会救助、社会福利和慈善事业等保障措施相互衔接、相互补充，使保障对象和制度做到不重不漏。切实加大保障性住房建设力度，积极实施廉租房、公共租赁房、经济适用房、限价商品房建设，加大城市棚户区改造力度，在市区陈仓、金陵湾等片区建设保障性住房110万平方米，在县城和重点镇建设保障性住房73万平方米。加快社会事业公共服务信息平台建设，并逐步向县乡村延伸，方便群众办事。

大力发展教育事业。全面启动“教育振兴八大工程”，促进教育现代化。加快学前教育发展，切实抓好乡镇中心幼儿园和社区幼儿园建设，缓解城乡“入园难”问题。加快中小学布局调整和标准化建设，促进义务教育均衡发展。加强宝鸡职业技术学院内涵能力建设，优化整合职业教育资源，加强“双师型”教师队伍和实训基地建设。加快市教育中心建设，建成市特殊教育学校。支持宝鸡文理学院建设硕士学位授予单位并筹建宝鸡综合性大学。

提升医疗卫生服务水平。加大公立医院改革力度，落实政府公共卫生补偿政策，优化医疗资源结构布局。贴息支持县以上等级医院提升医疗水平，推进城乡基层医疗机构标准化建设，有效缓解群众看病难问题。深入推进药品“三统一”工作，稳步实施国家基本药物制度，完善配套机制，调整提高报销比例，大力发展中医药，缓解群众看病贵问题。加强食品药品安全监管，确保食品药品安全。坚持“预防为主”方针，认真做好疾病预防控制、地方病防治、卫生监督和妇幼保健工作。深入开展“健康宝鸡”全民健康教育行动，巩固提升卫生县城（镇村）创建成果。

加快计生文化广电等社会事业发展。全面推进人口计生综合改革，深入开展农村母亲健康工程、优生促进工程，稳定低生育水平。加强基层文化体系建设，繁荣文化艺术创作，丰富群众文化生活，做好“非遗”申报和保护工作。加快市广电中心建设，基本完成广播电视“村村通”工程建设任务。积极申办省第十五届运动会，加快游泳跳水馆、射击中心建设步伐，办好全国乒乓球锦标赛等国家级赛事。支持社会力量兴建老年公寓、养老院、社区养老服务中心。加快推进城乡社区基础设施项目建设，扩大便民服务范围。为残疾人办好十件实事，创建全国残疾人工作示范城市。编制2011—2020年妇女儿童发展规划，为妇女儿童事业发展提供有力的政策保障。举全市之力创建全国文明城市。

深入推进社会管理创新。全面落实各级安全监管责任，加大对煤矿、非煤矿山、烟花爆竹、危险化学品、道路交通、建筑施工企业等高危行业的监管，完善各种应急预案，遏制重特大事故发生。全面实施社会稳定风险评估，完善“三调联动”机制，集中开展矛盾纠纷大排查、大调处工作，主动解决信访突出问题，及时发现并就地化解不稳定因素。启动实施“六五”普法规划，推进法治城市建设。以新一轮“平安宝鸡”创建活动为载体，扎实开展重点行业、重点领域、重点人群的严控严打，完善社区治安群防体系，提高社会治安管理水平。

（八）加强生态环境建设，不断提升可持续发展能力

切实加强节能减排。综合运用工程节能、技术节能和合同能源管理三项措施，全面推进工业、建筑、交通等重点领域节能。加强公共机构节能工作，完善建筑节能审批审查制度，为社会节能做好表率。加大企业技术改造力度，大力推广节能减排、清洁生产、污水及垃圾无害化处理、资源循环利用等技术，开展重金属污染防治工作，突出抓好水泥、化工、冶炼、造纸等重点企业的节能改造，加快淘汰落后生产能力。

加大环境保护力度。强化重点行业、重点区域污染整治，着力实施水污染、大气污染等专项整治行动，切实解决群众反映强烈的环境问题。全面启动渭河流域综合治理工程，加大千河流域生态环境治理力度，搞好污染防治，改善沿河两岸生态环境。大力实施生态治理工程，全面开展眉县霸王河、凤县小峪河等河流治理。做好迎接国家环保模范城市复检验收工作，争取国家生态示范市命名。实施村镇生活垃圾、污水处理、畜禽粪便处理示范项目，全市所有县区污水处理厂和垃圾处理场要达标运行。

深入推进绿色宝鸡建设。实施防护林建设，加强天然林保护，巩固退耕还林成果，广泛开展植树造林活动。积极实施城区绿化、台塬坡面绿化、交通道路绿化等“九大工程”，抓好渭河流域西山段和北坡绿化工作，完成植树造林1600万株。深入推进林权制度改革，在完善主体改革的基础上，加快推进配套改革。板块推进“三化一片林”生态家园示范村建设，扩大绿化面积。做好西安世园会宝鸡园筹建和参展工作。

四、全面加强政府自身建设

各位代表，形势的新变化和人民群众的新期待，对政府工作提出了更高的要求。各级政府必须加强自身建设，严格依法行政，切实转变职能，改进机关作风，真正做到对人民负责、受人民监督、让人民满意。

坚持科学决策。各级政府组成人员要加强学习，吃透中省政策，掌握市情特点，把握发展规律，找准结合点，既谋事又成事。坚持专家咨询、公众参与、社会公示、集体讨论等决策程序，提高决策的科学性。经常深入基层特别是问题较多的地方调查研究，问需于民，问计于民，问政于民。健全人民群众参与决策的途径和形式，提高政府发展经济、管理社会事务的能力和水平。

创新工作理念。大力推进思想观念、思维方式、工作方法创新，敢于打破常规，敢于实践尝试，用创新的理念发展经济、管理城市、富裕百姓。旗帜鲜明的鼓励创新，激励创造，推动创优，允许失败，使创新成为引领宝鸡发展的鲜明主题。主动学习沿海发达地区先进经验，在源头上缩小差距，在工作上不断追赶。用一流的规划和领先的创意设计引领宝鸡高水平发展，对涉及经济社会发展的重大事项，坚持规划设计高起点、高品位，每一个规划、每一项工作的谋划设计都要争取达到国内一流水平。

提高执行能力。各级政府和各部门“一把手”要围绕市委、市政府确定的重大事项、重点工作，跟踪督办，狠抓落实。盯住不落实的事，追究不落实的人，做到有责必问、有错必究，切实提高政府工作的执行力。不断优化发展环境，多服务、少干预，加强政务公开，推进依法行政，简化办事程序，提高办事效率。

用好财政资金。不断壮大骨干财源，大力培育新兴财源，切实增强财政实力。深入推进部门预算、国库集中支付、政府采购、非税收入征缴等制度改革，完善预算编制，把政府所有收支纳入预算管理，健全科学完整、结构优化、有机衔接、公开透明的政府预算体系。优化财政支出结构，更多地向农业、教育、医疗、住房、养老、社区服务等方面倾斜，全面提高公共服务的质量和水平。加强灾后重建工程决算和财政重点工程项目资金的审计监督，提高财政资金使用效益。

改进工作作风。各级政府要不断强化责任意识，敢于负责，勇于担当，遇事不怕、不躲、不推，做到对组织负责，对人民负责，对工作负责。各级公务员要以吃苦耐劳的工作作风，迎接各种竞争和挑战，勇于面对困难，敢于触及矛盾，以良好的作风取信于民，以实干的精神成就事业。增强做好群众工作的本领，密切联系群众，广泛了解社情民意，及时处理群众各种合理诉求，以实际行动赢得人民群众的尊重和拥护。

加强廉政建设。强化对权力运行的监督，规范各种行政事业性收费减免、土地征用出让、国有资产转让等公共资源权力运行，严格执行项目公开招投标制、经济责任审计制度，进一步规范行政行为和办事程序。各级政府的公务人员要严格按照《廉政准则》办事，严禁各级领导干部插手工程建设等领域经济活动。加强“三公”消费管理，压缩会议、接待、办公等公用经费，严格公务用车购置和管理，停止一般性因公出国出境。认真解决损害群众利益的问题和群众反映强烈的问题，严肃查处各类腐败问题，把政府廉政建设提高到一个新水平。

各位代表，站在新的起点上，实现新的跨越，是时代赋予我们的光荣使命。我们要在市委的坚强领导下，认真贯彻落实科学发展观，振奋精神，开拓创新，奋力实现“十二五”经济社会发展高点起步、良好开局，以优异的成绩迎接建党90周年！
